# Efficacy of leadless left ventricular endocardial stimulation for non-responders to cardiac resynchronization therapy

**DOI:** 10.1007/s10840-025-02155-z

**Published:** 2025-10-14

**Authors:** Nadeev Wijesuriya, Felicity De Vere, Sandra Howell, Alphonsus Liew, Baldeep Sidhu, Paolo Bosco, Prashanthan Sanders, Jagmeet P. Singh, Mary Norine Walsh, Steven A. Niederer, Christopher A. Rinaldi

**Affiliations:** 1 King’s College, London, UK; 2 Guy’s and St Thomas’s NHS Foundation Trust, London, UK; 3 University of Adelaide, Adelaide, Australia; 4 Massachusetts General Hospital, Harvard Medical School, Boston, MA USA; 5 Ascension St. Vincent Heart Center, Indianapolis, IN USA; 6 National Heart and Lung Institute, Imperial College London, London, UK; 7 Alan Turing Institute, London, UK; 8 Rayne Institute, St Thomas’s Hospital, 4th Floor Lambeth Wing, Westminster Bridge Road, SE1 7EH London, UK

**Keywords:** Leadless pacing, Cardiac resynchronization therapy, CRT non-response, Endocardial pacing, Heart failure

## Abstract

**Background:**

Non-responders to conventional coronary sinus CRT represent a high-risk heart failure population with limited treatment options. Leadless LV endocardial pacing may provide benefit by delivering more physiological activation patterns, and enabling pacing from optimized LV cavity sites. We aimed to evaluate the efficacy of leadless endocardial pacing using the WiSE-CRT system in CRT non-responders.

**Methods:**

A pooled patient-level post-hoc analysis of the SELECT-LV study, the WiSE Post-Market Registry and the SOLVE-CRT trial was performed. Multivariable regression analysis identified predictors of response to WiSE-CRT, followed by ROC analysis and subgroup analysis by degree of QRS narrowing.

**Results:**

The total cohort comprised of 71 patients. Complete echocardiographic data was available in 66 patients, and complete ECG data available in 45 patients. The clinical response rate (improvement in at least one NYHA class) was 46%. The “combined echocardiographic response rate” (composite of reduction in LV end-systolic volume of ≥15% or improvement in LV ejection fraction of ≥5%) was 51%. The degree of QRS reduction with WiSE-CRT compared to standard CRT was an independent predictor of combined echocardiographic response. ROC analysis generated an AUC of 0.68, with a Youden index cut-off of 19ms reduction in QRS. Subgroup analysis revealed a combined echocardiographic response rate of 70.5% above the cut-off and 38.5% below *p* = 0.04).

**Conclusion:**

Leadless endocardial CRT is a viable treatment option for conventional CRT non-responders. The degree of QRS narrowing with LV endocardial stimulation is predictive of response. Optimizing QRS reduction when testing LV pacing sites may be beneficial in maximizing outcomes.

**Graphical Abstract:**

**Supplementary Information:**

The online version contains supplementary material available at https://doi.org/10.1007/s10840-025-02155-z.

**Figure:**
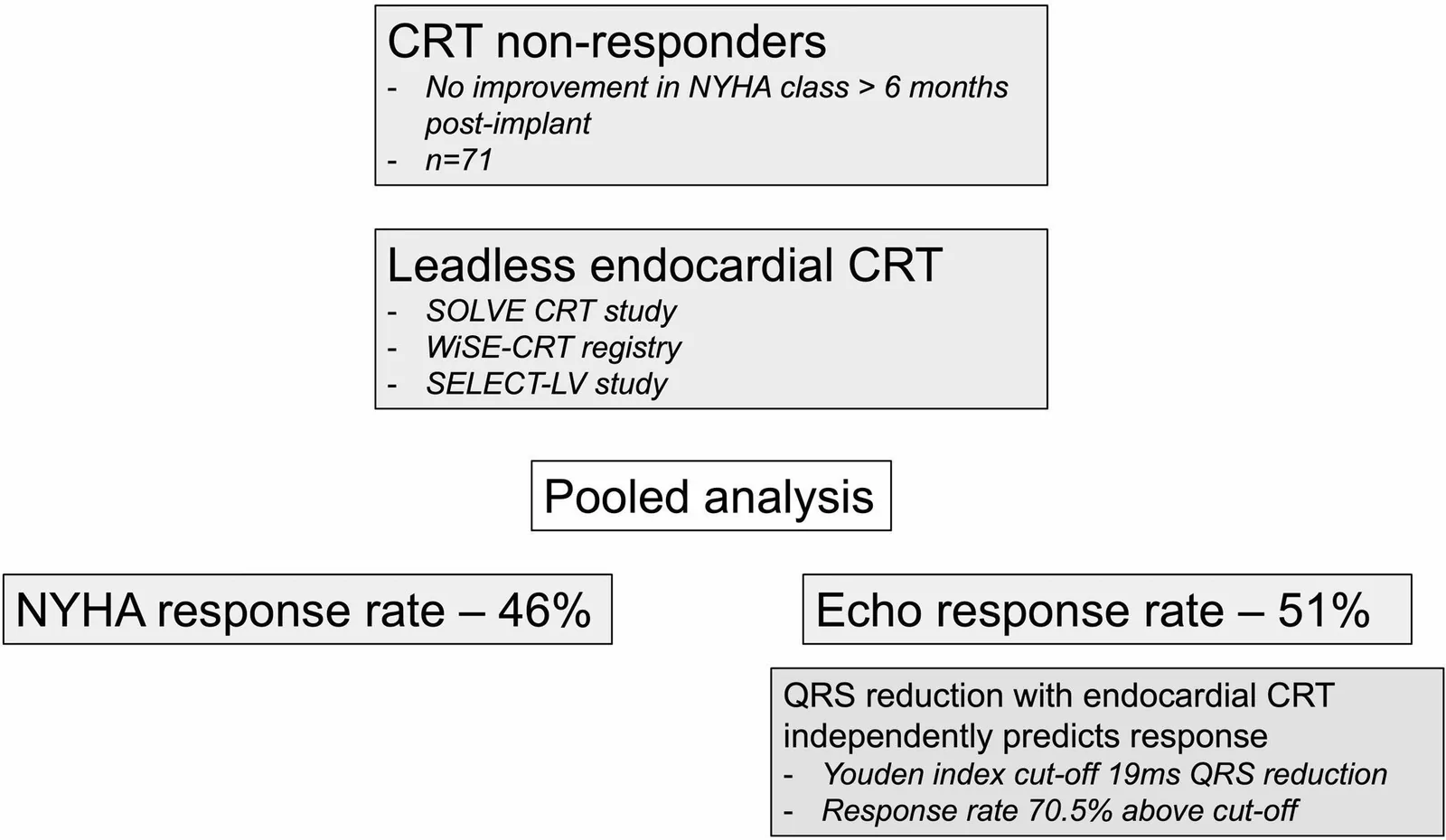

## Introduction

While cardiac resynchronization therapy (CRT) is an effective treatment for patients with heart failure (HF) and ventricular dyssynchrony, [1] 30–50% of patients do not respond to standard biventricular (BiV) pacing with a transvenous epicardial left ventricular (LV) lead [2]. CRT non-responders, as these patients are often deemed, are a high-risk, difficult to treat cohort of patients, who have limited options and have some of the poorest mortality outcomes of any heart failure sub-populations [3]. 

Several factors contribute to the limited efficacy of conventional CRT. Cardiac venous anatomy significantly restricts the pacing location of the LV lead, which may lead to difficulty in targeting optimal sites, and avoiding areas of transmural scar [4]. LV scar is present in up to 40% of CRT candidates, and it predicts poor response [5–8]. 

Computational modelling studies have also demonstrated that conventional epicardial CRT does not replicate physiological activation across the endocardium and in some instances may be pro-arrhythmic [9]. As such, there has been continued interest in the role of endocardial pacing for CRT. Hemodynamic studies have consistently demonstrated the superiority of endocardial LV pacing compared to epicardial pacing [10–12], however the uptake of permanent systems has been limited by high rates of thromboembolism associated with leads in the systemic circulation [13]. 

The development of leadless endocardial pacing systems may represent a solution to these problems. The WiSE system (EBR Systems Inc., Sunnyvale CA) is the only commercially available leadless LV pacing system [14]. The system consists of a battery connected to an ultrasound transmitter, which is implanted subcutaneously at the 4th, 5th or 6th intercostal place, and the receiver electrode, which is implanted in the LV cavity via aortic or trans-septal access [15]. The system requires the patient to have a “co-implant” in-situ capable of producing continuous RV pacing, which can be either a conventional transvenous device, or a leadless RV pacemaker. The transmitter and battery detect an RV pacing pulse emitted by the co-implant. Upon detection of the RV pacing spike, the transmitter emits several ultrasound pulses to locate the receiver electrode. Once the transmitter is electronically optimally aligned, a longer ultrasound wave is emitted, which is detected and converted to a pacing stimulus by the receiver electrode. This results in LV pacing, and thereby near simultaneous BiV pacing. Several studies, including the recent SOLVE-CRT trial, have demonstrated the safety and efficacy of this system in treating CRT eligible patients [16–18]. 

The WiSE device is approved in those patients who cannot receive or have failed conventional transvenous CRT implants due to issues such as venous obstruction (more common in upgrade patients), unfavourable coronary sinus branch anatomy, or high infection risk [19]. It has also been evaluated in those patients who are CRT non-responders, who theoretically may benefit from endocardial rather than epicardial pacing. Our group has previously reported in a cohort of 18 non-responders that treatment with WiSE led to an echocardiographic response rate of over 60% [20].

We aimed to re-examine the efficacy of WiSE treatment in a larger cohort of patients who were classified as non-responders, to assess whether leadless CRT is a viable second-line option for this high-risk patient population.

## Methods

**Fig. 1:**
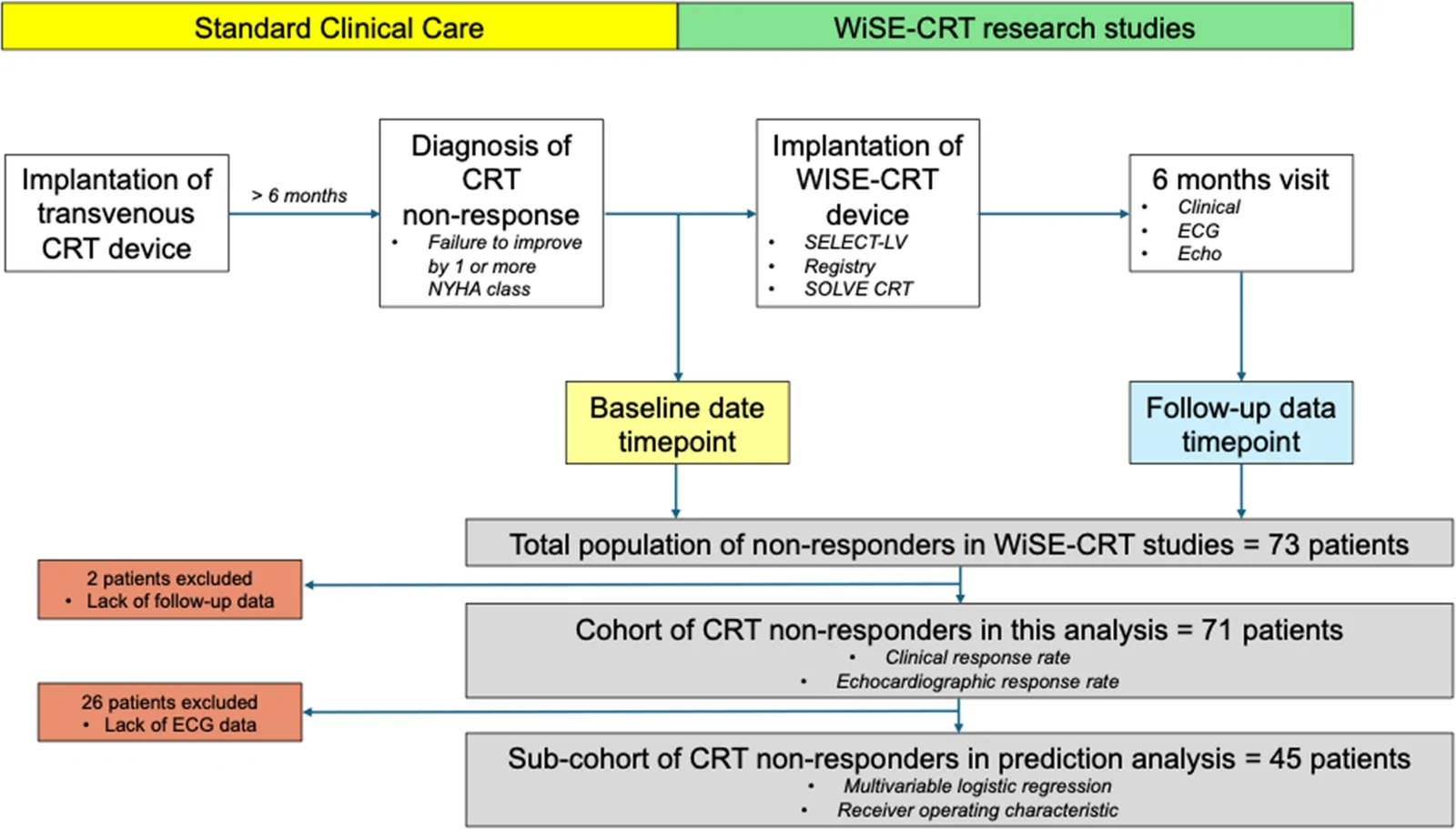
Patient flow chart from recruitment to leadless CRT studies to follow-up, timepoints of data collection and derivation of final analytic cohorts

We performed a pooled post-hoc patient-level analysis of two observational studies: the SELECT-LV study, [16] and the WiSE-CRT Post-Market Registry [17] We also included the pivotal SOLVE-CRT trial [18] which originated as a randomized trial that included patients in the non-responder category but was changed to a non-randomized design during to the COVID pandemic. Patients were included in this analysis if the indication for WiSE was non-response, defined as no change or worsening of New York Heart Association (NYHA) functional class after at least 6 months of conventional CRT (including AV-VV optimization), guideline directed medical therapy and after correction of reversible causes such as anemia or low BiV pacing. BiV pacing percentage was assessed via device interrogations. The three parent studies had been approved by relevant institutional review boards. The current sub-analysis did not involve any new patient recruitment or additional procedures beyond the original study protocol. A flow-chart illustrating data collection and integration is shown in Fig. 1.

### Implantation procedure

**Fig. 2:**
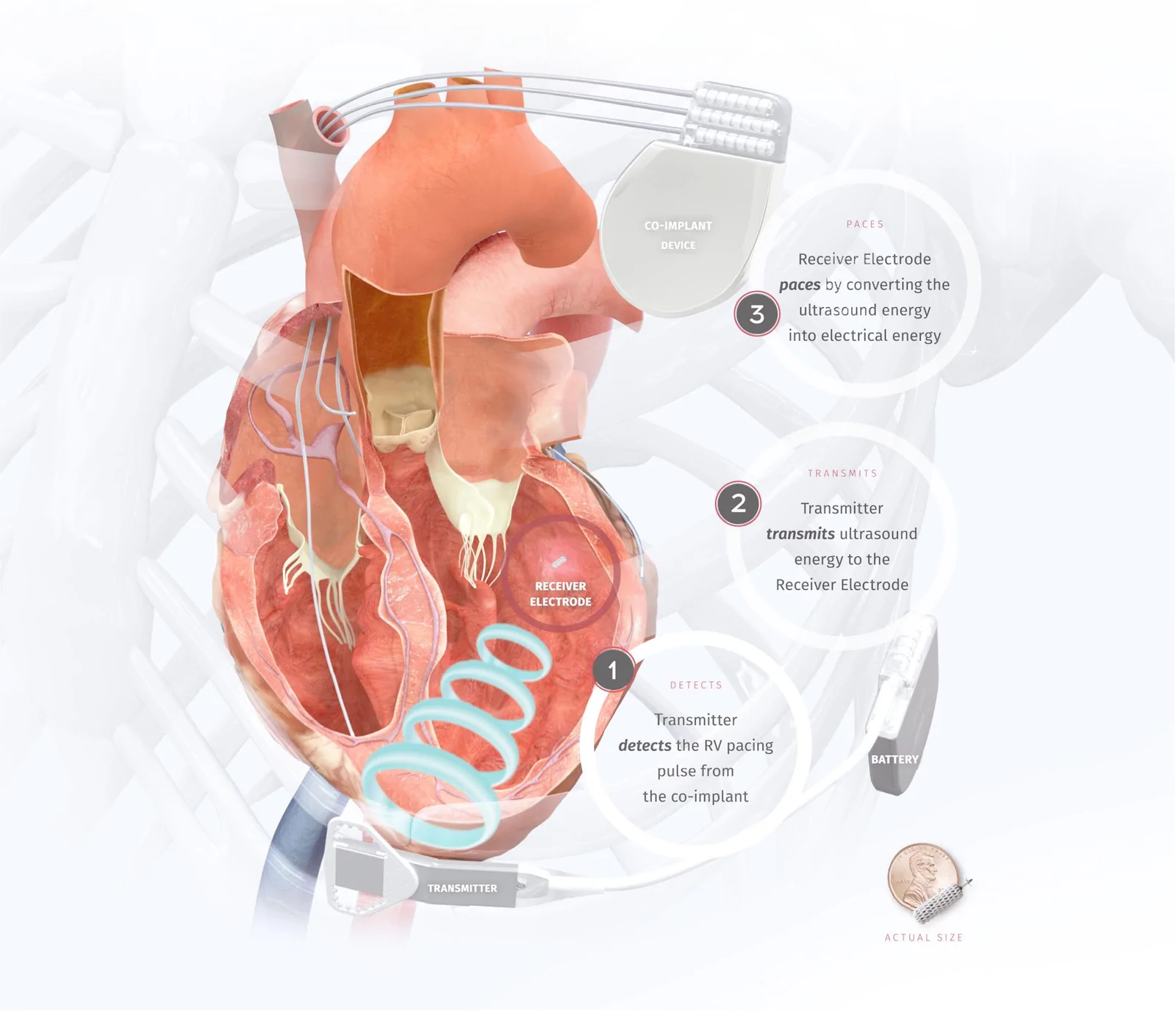
Components of the WiSE-CRT system. Battery and ultrasound transmitter implanted subcutaneously, with transmitter in 4th −6th intercostal space. The receiver electrode is implanted via femoral access, either via retrograde aortic or antegrade trans-septal approach into left ventricular (LV) cavity. In this figure, the electrode is implanted in the LV lateral wall. This can be done either at the time of the battery/transmitter implant (single-stage WiSE-CRT implant), or at a later date (dual-stage WiSE CRT implant). The co-implant device can be a conventional transvenous pacemaker, implantable cardioverter defibrillator (ICD), or a leadless device generating right ventricular (RV) pacing, which must have been implanted at least 6 weeks prior to the WiSE-CRT system. Reproduced from Wijesuriya et al., with permission [21]

All eligible patients had a standardized implantation of the WiSE device [14]. Patients underwent ultrasonic acoustic window screening to identify potential intercostal spaces for placement of the transmitter. During the first procedure stage the transmitter is placed within a preidentified intercostal space by dissecting to the intercostal muscle and confirming using ultrasound the adequacy of the window to the LV endocardial pacing site. The transmitter is then sutured into place with the battery placed in the mid-axillary line. During the second stage of the procedure, the endocardial electrode is inserted either via a retrograde aortic or trans-septal approach. The electrode is deployed within a segment in the LV, primarily on the lateral wall, based on factors such as sheath stability, acute QRS narrowing, electrical latency (QLV), and the location of any myocardial scar. In current practice, these factors are considered at physician discretion rather than in a standardized manner. A schematic for the device is illustrated in Fig. 2. The only clinically programmable setting for the device in BiV pacing, with LV pacing triggering off RV pacing stimulus, thus RV slightly ahead. The existing transvenous epicardial LV lead is switched off.

### Study endpoints

Efficacy endpoints in the studies were measured at 6 months post-implant.

1. Clinical response was defined as an improvement in NYHA functional class of 1 or more from baseline.
2. Echocardiographic response was defined as a reduction in LV end-systolic volume (LVESV) of greater than 15% *or* an increase in LV ejection fraction of greater than 5%. Echocardiographic indices were obtained using the Simpson’s Biplane Method [20, 22].

Echocardiographic images were independently analysed by a Core Laboratory in the SOLVE-CRT trial, but not in the SELECT-LV study or the Post-Market Registry.

### Statistics

Summary statistics were used for baseline characteristics. Continuous variables were tested for normality using the Shapiro-Wilks test and summarized by means and standard deviation (SD) if normally distributed, and by median and interquartile range if not. Discrete variables are expressed as count (%). Baseline characteristics between the subgroups for continuous variables were compared using a student’s t test if normally distributed and a Mann-Witney test if not. χ^2^ was used for discrete variables.

Multivariable logistic regression analysis was performed to identify the independent associates of WiSE combined echocardiographic response. The following baseline variables were selected a priori based on factors which are known to influence CRT response: age; sex; ischemic versus non-ischemic cardiomyopathy; hypertension; epicardial CRT QRS duration; underlying QRS (LBBB/RV paced versus non-LBBB); LV end-diastolic volume; QRS reduction with endocardial pacing. “QRS reduction” was defined as QRS duration with standard transvenous epicardial BiV CRT *minus* QRS duration with WiSE-CRT.

The Hosmer–Lemeshow Test was used to test the goodness-of-fit for the logistic regression model. Receiver Operating Characteristic (ROC) curves were generated for variables identified as significantly predictive on multivariable regression.

Analyses were conducted with Stata Statistical Software Package Release 18 (StataCorp LLC, College Station, TX). A p value of less than 0.05 was considered significant.

## Results

### Baseline characteristics

A total of 73 non-responder patients met initial screening criteria. Two patients were excluded due to lack of any follow-up data, leaving a final cohort of 71 patients. The baseline characteristics are shown in Table 1. The mean patient age was 68±8 years, with 76% being male. 48% had an ischemic cardiomyopathy phenotype. Patients had symptomatic HF with a baseline functional status of NYHA II (32%) or III (68%). The mean LVEDV was 209.3±69 ml (162.3±53 ml in females, 211.7±58 in males), and the mean LVEF was 26.5±7%. The baseline programmed CRT QRS duration was 152.1±26ms. 71% of patients had underlying LBBB or RV paced QRS morphology as the primary CRT indication. For those with an RV paced “underlying morphology”, this refers to having no underlying rhythm at VVI 30, and thus meeting criteria for a class 1 CRT indication for systolic dysfunction and pacing dependency. The median duration of treatment with standard CRT preceding the WiSE-CRT implant was 35 months (range 17–62). Five patients (7%) had received standard CRT implants in the preceding 6–12 months. Overall, this represents a cohort of symptomatic patients with increased LV dimensions, and severely reduced ejection fraction despite prolonged treatment with conventional CRT.

**Table 1:** Baseline characteristics. ^a^Data available in 70 patients ^b^Data available in 66 patients. ^c^Data available in 69 patients. ^d^Data available in 65 patients. RV paced underlying morphology refers to those who have no underlying rhythm at VVI 30

Variable	Total cohort (*n* = 71)
Age (years, mean±SD)	68±8
Sex	Male – 54 (76%) Female – 17 (24%)
Cardiomyopathy phenotype	Ischaemic – 34 (48%) Non-ischaemic – 37 (52%)
NYHA class	I – 0 (0%) II – 23 (32%) III – 48 (68%) IV – 0 (0%)
Hypertension ^a^	35 (50%)
Diabetes	30 (42%)
Device	CRT-P – 6(8%) CRT-D – 65(92%)
LV End-Diastolic Volume (ml, mean±SD) ^b^	209.3±65
LV ejection fraction (%, mean±SD) ^c^	26.5±7
Programmed CRT QRS duration(ms, mean±SD)	152.1±26
CRT indication^d^	Class I - LBBB, or RV pacing dependent – 47 (71%) Class II - Non-LBBB – 18 (29%)
Treatment duration of conventional CRT (months, median, IQR)	35 (17–62)

### Response rates

After implantation of the WiSE system, the overall clinical response rate, as assessed by an improvement in NYHA Class, was 46%. The mean reduction in LVESV (*n* = 62) was 8±16% (*p* < 0.01). The LVESV response rate (proportion of patients with reduction in LVESV greater than 15%) was 27%. The mean LVEF increase (*n* = 66) was 4.8±6% (*p* < 0.01). The LVEF response rate (proportion of patients with an improvement in LVEF of greater than 5%) was 42%. The combined echocardiographic response rate (LVESV reduction greater than 15% *or* LVEF improvement greater than 5%) in 67 patients with follow-up echocardiographic data was 51% (34 patients). Thirteen patients (20%) had an improvement in LVEF of greater than 10%. There were 30 patients exhibiting a positive combined echocardiographic response who had LV volumetric data available (the remaining 4 patients were classified as a responder based on LVEF improvement alone). In these 30 patients, there was a discrepancy between LVEF response and LVESV response in 19 (63%), Six patients exhibited a LVESV response without LVEF response > 5%, and 13 patients exhibited an LVEF response without an LVESV response.

**Fig. 3:**
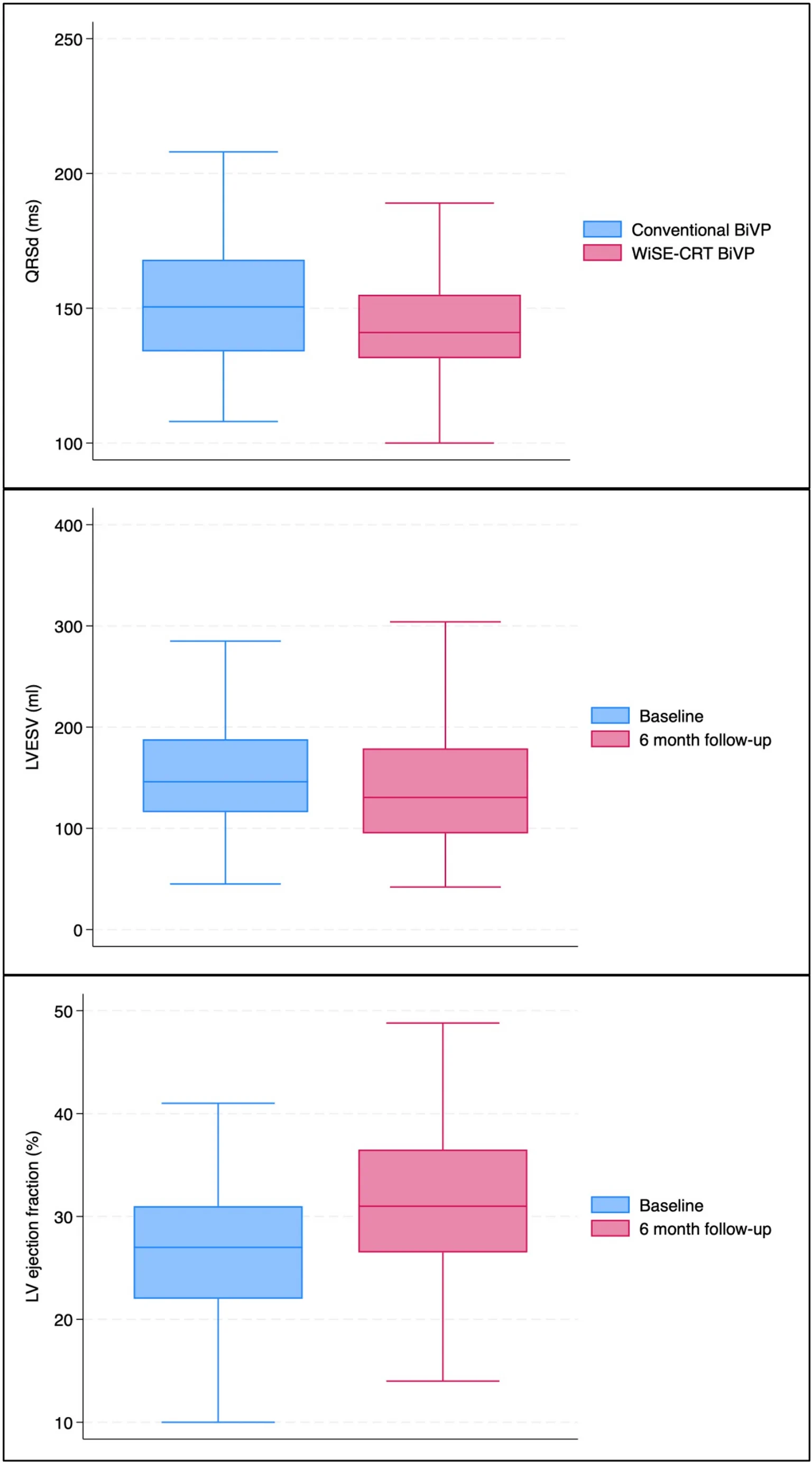
Box plots displaying changes in QRS duration between conventional CRT and WiSE-CRT (Panel **A**); change in LV end systolic volume from baseline to 6 months post WiSE-CRT implant (Panel **B**); and change in LV ejection fraction from baseline to 6 months post WiSE-CRT implant (Panel **C**). * denotes a significant difference, that is *p* < 0.05, between baseline and post WiSE-CRT

The mean reduction in QRSd, defined as QRSd with conventional CRT minus QRSd with WiSE-CRT (*n* = 45) was 11±28ms (*p* = 0.01). The echocardiographic and ECG data is summarized in Fig. 3.

### Deterioration rates

Six patients (8%) exhibited a “clinical deterioration”, that is, worsening of at least one NYHA class at follow up. In these patients, the mean increase in LVEF was.

−3.5%±4.7, compared to + 5.7%±6.3 (*p* < 0.01) in patients who stabilised or improved (that is, no change in NYHA class, or improvement in NYHA class).

### Predictors of wise echocardiographic response

Multivariable logistic regression analysis of combined echocardiographic response as a binary dependent variable (*n* = 45 with complete ECG data, baseline characteristics of this subgroup in Supplementary Table S1) revealed that increased QRS narrowing with WiSE compared to standard BiV CRT was a significant positive predictor (Table 2). The Hosmer–Lemeshow Test confirmed goodness-of-fit (*p* = 0.15). The univariate analysis is shown in Supplementary Table S2.

**Table 2:** Multivariable logistic regression analysis of predictors of combined echocardiographic response endpoint with WiSE-CRT treatment

Baseline variable	Odds ratio	Standard error	z	*P* value	95% CI
Age	0.926	0.057	−1.23	0.22	0.819–1.046
Male versus Female	0.358	0.343	−1.07	0.28	0.055–2.340
Ischaemic versus Non-Ischaemic	1.209	1.022	0.22	0.82	0.230–6.341
Hypertension	2.799	2.337	1.23	0.21	0.544–14.386
LBBB versus non-LBBB	0.383	0.355	−1.03	0.30	0.062–2.355
LV End Diastolic Volume	0.999	0.007	−0.11	0.92	0.985–1.013
QRS duration (ms)	0.987	0.021	−0.59	0.56	1.001–1.090
QRS reduction with WiSE-CRT (ms)	1.044	0.022	2.05	0.04	0.997–1.052

**Fig. 4:**
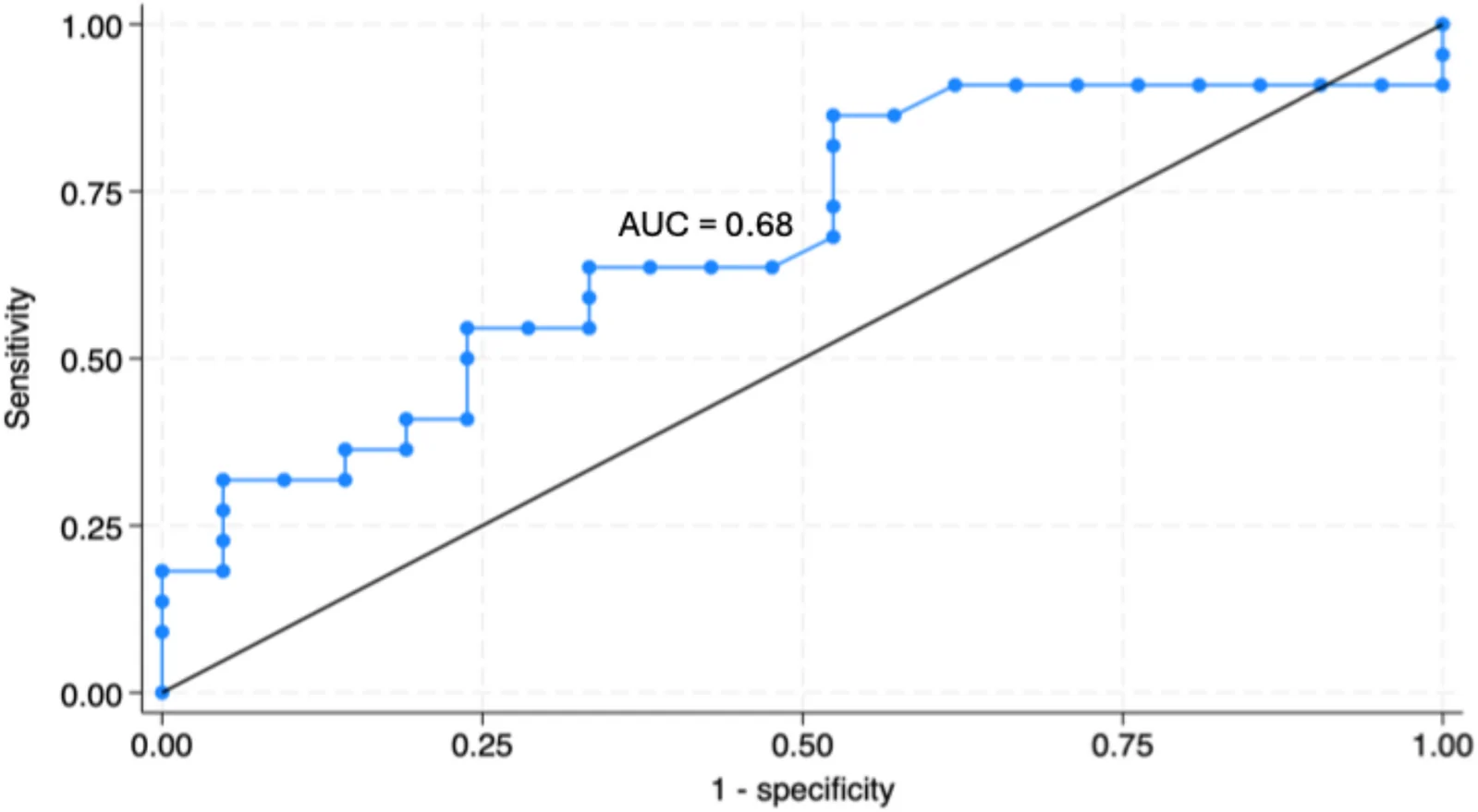
Receiver Operating Characteristic (ROC) curve. Combined echocardiographic response is the reference variable, QRS reduction with WiSE-CRT is the classification variable

A ROC Curve illustrating the performance of QRS narrowing at predicting WiSE combined echocardiographic response is shown in Fig. 4. The Area Under the Curve is 0.68, with the Youden Index cut-off being 19ms.

**Fig. 5:**
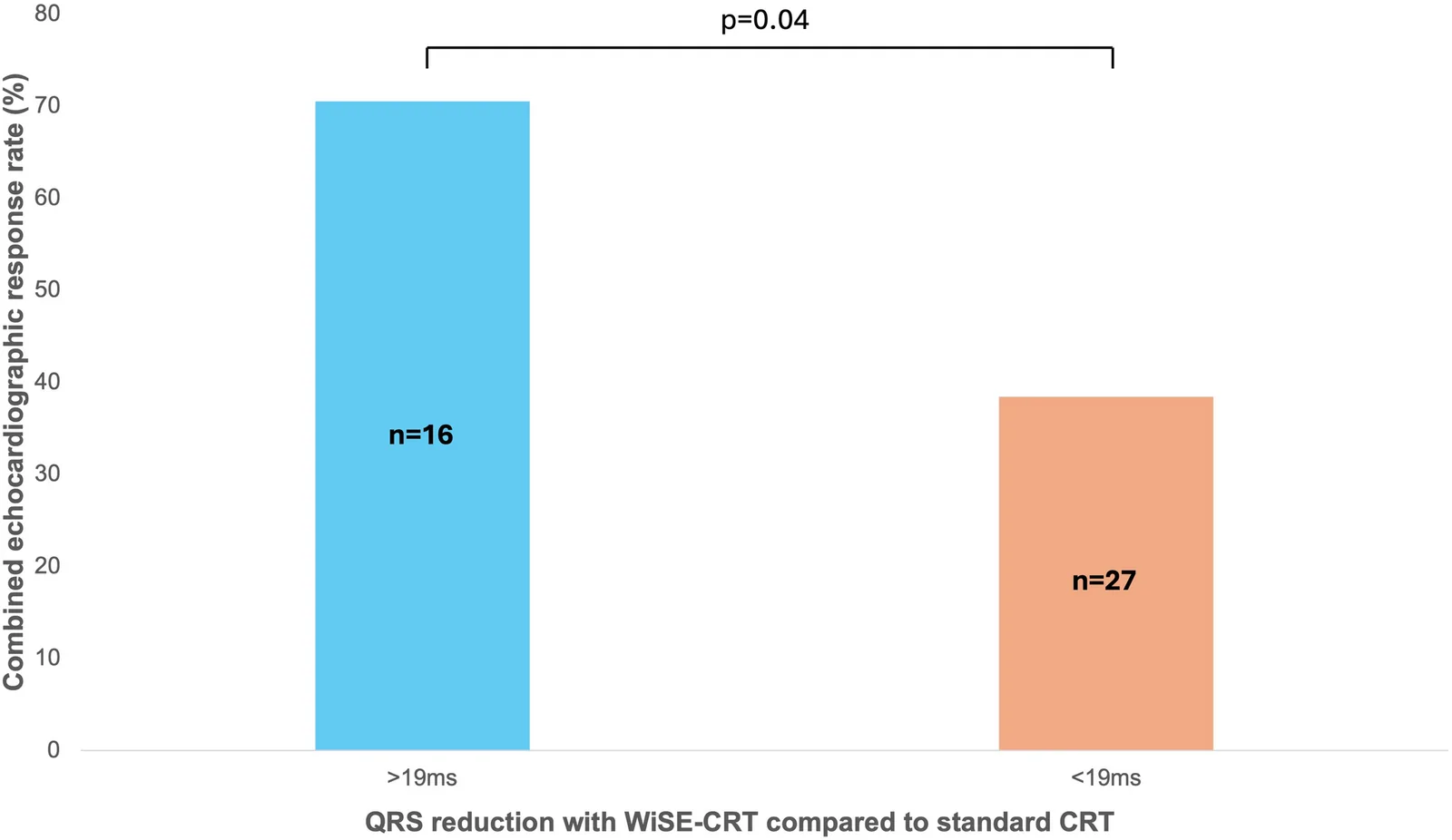
WiSE-CRT combined echocardiographic response as a function of QRS narrowing, with the cut-off generated as 19ms by Youden’s Index

Applying this cut-off, the echocardiographic response rate of patients with WiSE QRS narrowing of greater than or equal to 19ms was 70.5%, compared to 38.4% if the QRS narrowing was less than 19ms (*p* = 0.04, Fig. 5). The baseline BiV epicardial paced QRS duration was significantly longer in the group that achieved a QRS narrowing of > 19ms with WiSE (163.1ms±26 versus 143.5±21, *p* = 0.02).

There were 26 patients without complete ECG data who were not included in regression analysis. A comparison between the baseline characteristics of the “ECG available” versus “ECG not available” cohort is shown in Supplementary Table S1, with the only significant difference being the prevalence of hypertension being higher in the “ECG available” cohort. The combined echocardiographic response rate was 51% in the “ECG available” cohort versus 50% in the “ECG not available” cohort (*p* = 0.860).

## Discussion

We present the largest analysis, to date, examining the efficacy of leadless LV endocardial pacing as a treatment for non-responders to conventional CRT. Our findings were as follows:

1. The overall clinical response rate (as defined by an NYHA class improvement of at least 1 functional class) was 46%.
2. The overall “combined echocardiographic response” rate, (as defined by either a reduction in LVESV of greater than 15% *or* an improvement in LVEF of greater than 5%) was 51%.
3. QRS narrowing with WiSE compared to standard CRT was a significant predictor of echocardiographic response. The response rate was over 70% in patients with a QRS reduction of greater than 19ms.

### Endocardial pacing in CRT non-response

Endocardial pacing theoretically provides more physiological activation than epicardial pacing [23]. Importantly, it benefits from unrestricted LV pacing locations, vital in patients with factors such as ischaemic scar or lack of optimal cardiac venous targets. Mechanistic studies have consistently reported acute hemodynamic benefits for endocardial pacing versus conventional CRT [10, 12, 24–26]. Behar et al. [10] reported from a total of 135 sites tested in 8 patients that acute hemodynamic response (AHR) was significantly greater when temporary pacing the same myocardial segment endocardially versus epicardially and resulted in a shorter paced QRS duration. Sohal et al. [25] reported in an acute hemodynamic study of 10 patients with conventional CRT devices that the optimal LV endocardial pacing site was in the same myocardial segment as the existing epicardial LV lead in only 4 patients. Padeletti et al. similarly reported in 11 subjects that the optimal LV endocardial site in each patient significantly improved AHR compared to conventional epicardial LV stimulation [27]. These studies suggest that in an unselected CRT population, endocardial pacing improves AHR, with the greatest benefits seen when targeting an optimal pacing location.

Similarly, endocardial pacing has been shown to improve AHR in the conventional CRT non-responder cohort. Van Gelder et al. [28] performed an acute hemodynamic study in 24 patients and found that the initial CRT system yielded an AHR ≥ 15%in 5 patients after A-V and V-V optimisation. Of the remaining 19 patients, who did not demonstrate a positive AHR with conventional CRT, 9 had an increase in AHR to ≥ 15% at the optimal endocardial LV pacing position.

Our group previously evaluated the WiSE system in non-responders in a cohort of 18 registry patients who were include in this current analysis [20]. Sidhu et al. reported that endocardial pacing resulted in a significant reduction in QRS duration and improvement in LVEF. Overall, 55.6% of patients had improvement in their clinical composite score and 66.7% demonstrated an echocardiographic response [20]. 

The current findings extend these results in a larger cohort of 71 patients with a clinical and echocardiographic response rate of approximately 50%. This represents a significant improvement from alternate solutions, where other interventions including AV-VV delay optimization or multipoint pacing, have thus far not demonstrated benefits in non-responders [29, 30]. 

### Predicting response to wise in conventional CRT non-responders

Multiple factors have been identified as negative predictors of response to conventional BiV CRT including male sex, arrhythmia and non-LBBB morphology [31]. Our analysis did not demonstrate this when examining which variables predict response to WiSE in conventional CRT non-responders with only QRS narrowing with WISE being predictive of conversion of non-responders to responders. It is possible that these patterns may have emerged with a larger patient cohort. However, there are multiple factors such as sub-optimal LV lead position; sub-optimal medical therapy, medication adherence, or primary RV dysfunction which are difficult to capture accurately in studies, but likely have a significant influence on CRT outcomes. It may be that the complexity and heterogeneity of non-responders means that identifying predictive baseline variables for WiSE-CRT treatment is more challenging.

Our study did identify a procedural variable, the degree of QRS narrowing with endocardial CRT compared to standard CRT, as borderline significantly predictive of WiSE response, with a modest AUC on ROC analysis. This variable likely identified the subgroup of patients where the primary etiology of non-response was persistent electrical mechanical dyssynchrony despite standard BiV pacing. These are likely to be the patients who will benefit the most from the alternative pacing location delivered by leadless CRT, where synchrony can be maximised by identifying an optimal site for the LV electrode implantation. The finding that *QRS reduction* with WiSE-CRT, not *baseline QRS duration* pre WiSE-CRT, was predictive suggests that finding the optimal pacing site is likely to bear importance in the outcome of endocardial pacing – identification of patients with ongoing dyssynchrony is just the first step, the next being performing resynchronization successfully where standard epicardial CRT was not. Several methods have been identified as having potential for guiding WiSE implants, including standard intra-procedural methods such as Q-LV [32] and advanced pre-procedural planning using perfusion CT to identify the latest activating myocardial segments [33]. 

### Clinical application

Currently, there are limited options for the treatment of CRT non-responders, a high-risk and generally highly co-morbid patient cohort. Interventions such as AV optimization and multipoint pacing have not demonstrated sizeable benefits in these patients. Conduction system pacing and more advanced modalities such as Left Bundle Optimized CRT (LOT-CRT) [34] have shown improved acute electrical synchrony compared to standard CRT. In a multicentre observational study, Vijayaraman et al. reported that echocardiographic response (LVEF improvement of >5%) was seen in 40% of patients who received left bundle branch area pacing (LBBAP) as rescue therapy for conventional CRT non-response. In their cohort, the clinical response rate was 45% [3].

Leadless endocardial CRT appears to have a similar efficacy to that reported from LBBAP rescue therapy, with this study demonstrating both a clinical and echocardiographic response rate of approximately 50%. It should be noted that as a novel therapy, leadless CRT has a higher procedural complexity and complication rate than transvenous LBBAP, with a type 1 complication rate of 19% reported in the SOLVE-CRT study [18]. In the absence of prospective randomised data comparing efficacy of these two novel CRT modalities, in current clinical practice leadless CRT would be favoured if there are factors prohibiting a relatively “simple” implantation of a further transvenous lead, for example blocked subclavian venous access, high infection risks or transmural septal scar.

Data thus far has not revealed baseline factors which may predict which patients will derive benefits from WiSE-CRT, however, it those with higher QRS narrowing with LV endocardial pacing appear more likely to respond. It may be that patients with persistent electrical dyssynchrony, that is, QRS widening despite standard epicardial CRT, will stand to benefit most from LV endocardial stimulation, provided that an endocardial site with improved resynchronization is found. There may also be scope for endocardial resynchronisation to improve further if the technology is updated to allow V-V optimisation. In its current form, WiSE-CRT provides BiV pacing with RV slightly ahead, which is very rarely the optimal setting in the context of conventional CRT. Refinements to allow pacing with LV ahead, for example, timing from an atrial rather than RV impulse, may be useful in maximising device benefits.

### Limitations

This is a pooled patient-level post-hoc analysis of three studies, and as such has inherent limitations of selection bias.

In the first instance, the definition of CRT non-response used when recruiting for the WiSE-CRT studies was derived from NYHA response. As such, our findings may not necessarily translate to patients who meet alternative definitions of non-response e.g. LVESV or LVEF non-response. On a related note, the initial CRT indication in this patient population was heterogenous. This is the nature of the current state of leadless CRT - for example, in the WiSE Post Market Registry, 90% of patients were those with systolic dysfunction and RV pacing dependency rather than the more traditional LBBB cohort. In our cohort, LBBB and RV pacing dependency were reported as pooled characteristic as these both represent a class I CRT indication.

In terms of data, whilst the total dataset was 71 patients, complete ECG data was available in 45 patients, thus limiting the size of the regression and QRS subgroup analysis, and this must be taken into account when considering the strength of the conclusion. We presented a comparison of baseline characteristics and echocardiographic outcomes of the “ECG available” versus “ECG not available” cohorts in an effort to examine for evidence of selection bias.

Complete echocardiographic data, including LV volumes, was available in 62 patients. As such, the primary efficacy outcome analysed was “combined echocardiographic response” defined by either a reduction in LVESV greater than 15% or an improvement in LVEF of greater than 5%. This outcome was utilised as: (1) it was a metric which encompassed all patients with echo data available for inclusion in the analysis; and (2) this was the outcome utilised in our previous smaller study of WiSE efficacy in non-responders [20], and in prior studies of conventional CRT [22]. This is a relatively lenient definition of CRT response, however it aims to capture all patients who may derive a clinically meaningful improvement with second line CRT therapy. It should be noted that at present, there is little evidence validating the definition of “response” to second line CRT therapy, where significant volumetric changes may not be as readily observed compared to de novo implants. In their study of LBBAP as a rescue therapy following conventional CRT non-response, Vijayaraman et al. used LVEF increase of 5% as the definition of response [3]. 

For transparency and completeness have reported in our results the response rate when the more conventional outcome of reduction in LVESV greater than 15% is applied (the primary outcome in the SOLVE-CRT trial), and this gives more modest estimates of clinical benefit. We also report in the results the discrepancy rate between volumetric and LVEF response, with this being the case in 19 out of 30 patients where both parameters were measured. This discrepancy may highlight how volumetric changes in particular are likely to be more modest with second line therapy.

Of note, contemporary thinking is that CRT response should not be thought of as a dichotomous event, but rather a spectrum of outcomes including LV reverse remodelling, improvement in systolic contraction, delayed improvements, and stabilization of function [35]. Our reporting or multiple outcomes reflects this notion.

In addition, baseline dyssynchrony markers such as apical rocking or septal flash were not collected in these studies. Their inclusion in future studies may help to refine prediction models. We also acknowledge additional data which was not available in this analysis, but should be considered as important in further prospective studies in this area. In terms of baseline and procedural characteristics, this would be: AF; pre- and post-CRT ECG findings (classed in this study as LBBB or RV paced versus non-LBBB); _epicardial lead position and final WiSE electrode position. Programming parameters for the original CRT device, in particular, if CRT optimisation was performed and if so by which method, was not available in this study. On a related note, there was no data on whether methods such as adaptive LV only pacing were utilised rather than standard BiV pacing. It is known that suboptimal device settings may be contributory to non-response in a significant number of patients, and as such we cannot exclude that further QRS reductions could have been achieved with conventional CRT applying better programming, as this was not specified in the protocol [36]. 

Furthermore, assessment of LVEF improvement in relation to *original* LVEF (i.e. prior to transvenous device implant) would also ideally be examined, however this data was not collected in the parent studies. This is one of the limitations of studying non-responders as a subgroup of larger studies where the majority of patients are receiving de novo CRT – there may be pertinent data which is not captured by the standard case report form.

Despite incomplete data, this is the largest study of WiSE in non-responders to date. The three studies analysed were prospective, with standardized WiSE implantation protocols and follow-up echocardiography procedures. Echocardiographic data was assessed by a core laboratory in the SOLVE-CRT study, which contributed 54 out of 71 patients in this cohort (echocardiograms were not core lab adjudicated in the SELECT-LV study or Registry). Study monitoring was performed with source data verification. Whilst there was no control arm in this study, the mean time of WiSE-CRT implantation post conventional CRT was approximately 3 years. This to an extent mitigates the potential confounding bias from so called “delayed response” to CRT, where a cohort of patients will show evidence of LV reverse remodelling after the standard follow-up endpoint of 6 months, usually between 6 and 12 months [30]. 

The current analysis addresses an important issue of CRT non-response. Our findings should be considered hypothesis generating, and a randomized control trial would be required to further evaluate of the use this technology in non-responders, with more detailed data collection targeted to examine non-response, and inclusion of procedural factors such as LV lead and LV electrode positioning and the effect on outcomes.

## Conclusion

Treatment with leadless endocardial CRT in conventional CRT non-responders resulted in a 51% echocardiographic response rate. Greater QRS narrowing compared to standard CRT was predictive of response. Targeting optimal endocardial pacing sites may improve outcomes.”

## Supplementary Information

Below is the link to the electronic supplementary material.

Supplementary Material 1 (DOCX 17.9 KB)

## Data Availability

No datasets were generated or analysed during the current study.
