# Supplementary material for: Efficacy of leadless left ventricular endocardial stimulation for non-responders to cardiac resynchronization therapy
Source: J Interv Card Electrophysiol. 2025 Oct 14;69(7):1817–28. doi: 10.1007/s10840-025-02155-z (PMC13687124; doi:10.1007/s10840-025-02155-z)
Supplement: Supplementary file 1 [file 10840_2025_2155_MOESM1_ESM.docx]

**SUPPLEMENTARY MATERIAL**

| Variable | ECG cohort (n=45) | No ECG cohort (n=26) | Comparison |
| --- | --- | --- | --- |
| Age (years, mean±SD) | 68.2±7 | 67.6±9 | p=0.75 |
| Sex | Male – 34 (76%)  Female – 11 (24%) | Male – 20 (77%)  Female – 6 (23%) | p=0.89 |
| Cardiomyopathy phenotype | Ischaemic – 20 (45%)  Non-ischaemic – 25 (55%) | Ischaemic – 9 (35%)  Non-ischaemic – 17 (65%) | p=0.41 |
| NYHA class | I – 0 (0%)  II – 17 (38%)  III – 28 (62%)  IV – 0 (0%) | I – 0 (0%)  II – 6 (23%)  III – 20 (77%)  IV – 0 (0%) | p=0.20 |
| Hypertension | 28 (62%) | 7 (27%) | p=0.004 |
| Diabetes | 22 (48%) | 8 (31%) | p=0.13 |
| Device | CRT-P – 6 (13%)  CRT-D – 39 (87%) | CRT-P – 0 (0%)  CRT-D – 26 (100%) | p=0.18 |
| LV End-Diastolic Volume (ml, mean±SD) | 206.9±62 ^a^ | 214.3±72^c^ | p=0.64 |
| LV ejection fraction (%,mean±SD) | 27.3±5 | 25.2±8 | p=0.18 |
| Biventricular paced QRS duration(ms, mean±SD) | 151.3±24 | Not available |  |
| Underlying QRS morphology | LBBB or RV paced – 28 (65%)  Non-LBBB – 15 (35%)^b^ | LBBB or RV paced – 18 (81%)  Non-LBBB – 4 (19%)^c^ | p=0.16 |
| Treatment duration of conventional CRT (months, median, IQR) | 35 (17-62) | 39.7 (28-66) | p=0.31 |

*Table S1* – Baseline characteristics of patients with ECG follow-up data. These patients were included in the multivariable logistic regression analysis. This is compared to patients without ECG follow up data.

^a^Data available in 44 patients. ^b^Data available in 43 patients ^c^Data available in 22 patients.

| **Baseline variable** | **Odds ratio** | **Standard error** | **z** | **P value** | **95% CI** |
| --- | --- | --- | --- | --- | --- |
| Age | 0.953 | 0.030 | -1.5 | 0.13 | 0.896 – 1.014 |
| Male versus Female | 0.820 | 0.461 | -0.35 | 0.72 | 0.272 – 2.470 |
| Ischaemic versus Non-Ischaemic | 1.062 | 0.519 | 0.12 | 0.90 | 0.407 – 2.769 |
| Hypertension | 2.087 | 1.046 | 1.47 | 0.14 | 0.782 – 5.573 |
| LBBB versus non-LBBB | 0.600 | 0.335 | -0.91 | 0.36 | 0.201 – 1.795 |
| LV End Diastolic Volume | 1.002 | 0.003 | 0.76 | 0.44 | 0.995 – 1.010 |
| QRS duration (ms) | 1.006 | 0.111 | 0.58 | 0.56 | 0.984 – 1.029 |
| QRS reduction with  WiSE-CRT (ms) | 1.024 | 0.013 | 1.79 | 0.07 | 0.997 – 1.052 |

*Table S2* – Univariable logistic regression analysis of predictors of combined echocardiographic response endpoint with WiSE-CRT treatment.
